# Benefit Finding in Maintenance Haemodialysis Patients: Associations With Social Support and Life Orientation

**DOI:** 10.1002/nop2.70946

**Published:** 2026-10-28

**Authors:** Xianghao Chen, Xiaodan Lin, Haidan Xie, Jialian Huang, Huifeng Song, Xiaozhou Zhou

**Affiliations:** ^1^ School of Nursing Guangdong Pharmaceutical University Guangzhou China; ^2^ Department of Nephrology The First Affiliated Hospital of Guangdong Pharmaceutical University Guangzhou China; ^3^ Department of Blood Purification The First Affiliated Hospital of Guangzhou University of Chinese Medicine Guangzhou China; ^4^ Department of Nursing The First Affiliated Hospital of Guangdong Pharmaceutical University Guangzhou China

**Keywords:** benefit finding, haemodialysis, influencing factors, life orientation, social support

## Abstract

**Aim:**

To explore the current status of benefit finding (BF) in maintenance haemodialysis patients and identify its key influencing factors.

**Design:**

A cross‐sectional study.

**Methods:**

A total of 250 maintenance haemodialysis patients were recruited via convenience sampling from the blood purification centres of two Grade A tertiary hospitals between September 2024 and March 2025. Data were collected using a general information questionnaire, the BF Scale, the Perceived Social Support Scale and the Life Orientation Test‐Revised. One‐way analysis and multiple linear regression analysis were conducted to explore the associations and influencing factors of BF.

**Results:**

Benefit finding was positively correlated with social support (*r* = 0.423, *p* < 0.001) and life orientation (*r* = 0.236, *p* < 0.001). Female gender, higher education level, retired status, family support, friend support and life orientation were significant predictors of BF.

**Patient or Public Contribution:**

No patient or public involvement was conducted in this study.

**Implications for Nursing Practice:**

Nurses should routinely assess BF and related psychosocial factors in maintenance haemodialysis patients. Nurse‐led interventions that strengthen family and peer support and enhance optimism may help promote BF in this population.

## 1 Introduction

Maintenance haemodialysis (MHD) is an essential renal replacement therapy for individuals with end‐stage renal disease (ESRD), requiring regular blood purification to eliminate metabolic waste and surplus fluid from the body (Zhu, Wu, et al. 2024). Although MHD prolongs survival, prolonged treatment places considerable physical and financial strain on patients, resulting in psychological distress such as anxiety and depression, which substantially reduces their quality of life (Shdaifat et al. 2024).

As positive psychology has developed, there has been growing interest in the positive psychological resources of patients. Benefit finding (BF) refers to the cognitive process by which individuals identify positive meanings and opportunities for growth within adverse experiences (Taylor 1983). Evidence indicates that BF can mitigate the negative psychological effects of chronic illness and encourage the use of adaptive coping strategies (Liu et al. 2026).

Social support refers to the resources offered by others and can be divided into emotional, instrumental, informational and appraisal support (Styck et al. 2025). It is widely regarded as an important buffer against stress and a key contributor to positive adjustment among individuals with chronic illness. Life orientation, conceptualized as dispositional optimism, describes the general expectation that favourable outcomes will occur in the future (Biber et al. 2025). People with stronger dispositional optimism are more likely to use active coping strategies and to show better psychological outcomes when confronted with adversity.

In contrast to other chronic conditions, MHD patients encounter specific difficulties, such as lifelong treatment, restrictive dietary and fluid regimens and considerable financial pressure, all of which may shape their BF experiences in distinctive ways. Although BF has been examined in cancer and chronic obstructive pulmonary disease (COPD) populations (Zhang et al. 2024), studies focusing on MHD patients remain scarce.

Social support (Pei et al. 2025) and life orientation, conceptualized as dispositional optimism (Patel et al. 2025), are well‐established psychosocial factors that support positive adjustment in chronic illness populations. However, no study has yet examined their roles in BF simultaneously among MHD patients.

Earlier studies have reported that social support and optimism are positively associated with BF (Zhao et al. 2025) and that higher BF levels are linked to better quality of life, lower psychological distress and greater optimism (St Fleur et al. 2024). The influence of socio‐demographic factors such as age, education and income on BF remains uncertain.

Accordingly, this study aims to examine the current status of BF in MHD patients and to identify its influencing factors, with particular attention to the roles of social support and life orientation, so as to provide evidence for targeted interventions that promote patients' mental health.

## 2 The Study

### 2.1 Aim

The primary aim of this study is to explore the current status of BF among MHD patients. Additionally, this study aims to identify the key factors influencing BF in this population, particularly the associations between BF, social support and life orientation. Accordingly, the research questions are as follows: (1) What is the current status of BF among MHD patients? (2) What are the associations between BF and social support among MHD patients? (3) What are the associations between BF and life orientation among MHD patients? (4) What are the key factors influencing BF among MHD patients, particularly social support and life orientation?

## 3 Materials and Methods

### 3.1 Design and Sample

This is a cross‐sectional study. Convenience sampling was employed to recruit MHD patients from the blood purification centres of two Grade A tertiary hospitals between September 2024 and March 2025. Patients were recruited from both morning and afternoon dialysis shifts to ensure representation. Of the 268 eligible patients approached, 250 provided informed consent to participate, resulting in a response rate of 93.3%. Inclusion criteria: (i) Age ≥ 18 years; (ii) Diagnosed with ESRD according to the ‘Chronic Kidney Disease Screening, Diagnosis and Prevention Guidelines’ (Expert Group on Early Detection, Diagnosis and Treatment of Chronic Kidney Disease 2017); (iii) Regular dialysis treatment for ≥ 3 months; (iv) Basic literacy and comprehension skills in Chinese; (v) Informed consent and voluntary participation in this study. Exclusion criteria: (i) Simultaneous peritoneal dialysis; (ii) Temporary dialysis; (iii) Concurrent serious conditions such as heart failure, respiratory failure, pulmonary embolism, etc.; (iv) Individuals with severe mental illness or communication disorders. Sample size was estimated using G*Power 3.1 software. For a multiple linear regression with 10 predictors, a medium effect size (*f* ^2^ = 0.15), *α* = 0.05 and statistical power = 0.80, a minimum sample of 118 participants was required. Our final sample of 250 exceeds this requirement and is sufficient for the analysis.

### 3.2 Materials

#### 3.2.1 General Information Questionnaire

The researchers themselves designed factors such as gender, age, marital status, fertility status, family caregivers, education level, healthcare payment methods and types of medication.

#### 3.2.2 Benefit Finding Scale

The BF Scale was employed in the present study (Yan et al. 2023). The scale comprises 26 items across six dimensions: spiritual growth, appreciation of life and living, personal growth, appreciation of social support, altruistic behaviour and health behaviour change. Each item is rated on a 5‐point Likert scale ranging from 0 (‘not at all’) to 4 (‘extremely’), yielding total scores ranging from 0 to 104. Higher aggregate scores signify higher levels of BF among participants. In this study, the Cronbach's α coefficient of the scale was 0.812, indicating good internal consistency. Confirmatory factor analysis supported the original six‐factor structure, demonstrating satisfactory construct validity for this scale among patients with chronic conditions.

#### 3.2.3 Perceived Social Support Scale

This scale was originally developed by Zimet et al. (Zimet et al. 1990). The Chinese version was used in this study and has demonstrated good reliability and validity in Chinese populations. The scale is primarily used to measure an individual's perception of support from family, friends and others. It consists of 3 dimensions and 12 items, rated on a 7‐point Likert scale, with total scores ranging from 12 to 84. Higher scores indicate a stronger perception of social support. Specifically, scores between 12 and 36 indicate a low level of support, scores between 37 and 60 indicate a moderate level and scores between 61 and 84 indicate a high level. In this study, the Cronbach's α coefficient of the scale was 0.892, demonstrating excellent reliability.

#### 3.2.4 Life Orientation Test‐Revised

The life orientation test (LOT) was first developed in 1985 by Scheier and Carver (Scheier and Carver 1985) based on self‐regulation theory. Later, Scheier (Scheier et al. 1994) developed a revised version (LOT‐R). The scale is designed to assess an individual's dispositional optimism—that is, their tendency toward an optimistic personality. The LOT‐R is unidimensional and consists of 10 items, of which 4 are filler items and not scored. In this study, the 6 scored items were used, with responses rated on a 5‐point Likert scale and total scores ranging from 0 to 24. Higher scores reflect a more optimistic personality. In this study, the Cronbach's α coefficient of the scale was 0.746, indicating acceptable internal consistency. The Chinese version of the LOT‐R has been validated for use in mainland Chinese populations, demonstrating adequate psychometric properties.

### 3.3 Data Collection

The research data were collected through a standardized process. Data were collected by two trained postgraduate students to ensure consistency. First, approval was obtained from the hospital's ethics committee and the blood purification center management department. The researchers then screened potential participants against the inclusion and exclusion criteria, using a standardized script to explain the study purpose, principles of anonymity and confidentiality to the patients. Following informed consent, questionnaires were distributed and completed on site. For participants with visual impairment, motor impairment, or low literacy, the research team administered the questionnaire by reading each item aloud and recording the participants' responses verbatim. No interpretation was provided during this process. The completeness and accuracy of each questionnaire were verified immediately after completion. Ambiguous or missing responses were resolved through direct patient contact, in which the researchers confirmed the information and clarified relevant items via face‐to‐face discussion. Patients' diagnostic profiles and treatment parameters were cross‐referenced against the hospital's electronic medical record system. Two researchers independently performed data entry and validation to guarantee the authenticity and consistency of the collected data. The response rate was 93.3%. During data collection, the completeness and accuracy of each questionnaire were checked immediately upon completion. For ambiguous responses or missing items, the researchers asked the participants to confirm and correct them face‐to‐face. Eighteen questionnaires were identified as invalid because of patterned responding or obvious logical inconsistencies and were therefore excluded from the final analysis. As a result, no missing data remained in the final dataset and no imputation procedures were applied. The flow of participants through the study is shown in Figure S1. The STROBE checklist for cross‐sectional studies is provided as Data S1.

### 3.4 Statistical Analyses

All statistical analyses were performed using the Statistical Package for the Social Sciences (SPSS) version 27.0. First, a normality test was performed. Continuous data following a normal distribution were presented as mean ± standard deviation; categorical data were described using frequencies and percentages. For inter‐group comparisons, differences between two groups were analysed using an independent samples *t*‐test, while comparisons among multiple groups were conducted using ANOVA. When ANOVA indicated statistically significant differences, post hoc tests using the least significant difference (LSD) method were performed to identify specific group differences. In addition, multiple linear regression analysis was employed to explore the influencing factors of BF in MHD patients. A *p*‐value of < 0.05 was considered statistically significant.

## 4 Results

### 4.1 General Information of MHD Patients

A total of 250 valid patients were collected in this study, including 148 male patients, accounting for 59.20% of the total; the majority of participants (*n* = 106, 42.40%) were aged 45–60 years and most participants (*n* = 185, 74.00%) were married. Detailed demographic and clinical characteristics of all participants are shown in Table 1.

**TABLE 1:** BF in MHD patients grouped by demographic characteristics (*n* = 250).

Variable	*N*	%	BF score (points, $\bar{x}\pm s$)	*t*/*F*	*p*
Gender					
Male	148	59.2	53.72 ± 14.44	−2.143	0.033*
Female	102	40.8	57.74 ± 14.70		
Age(years)					
18–45	68	27.2	54.16 ± 15.52	1.709	0.183
45–60	106	42.4	57.34 ± 14.64		
> 60	76	30.4	53.67 ± 13.72		
Residence					
Town	200	80.0	56.03 ± 14.67	1.460	0.146
Rural	50	20.0	52.66 ± 14.43		
Marital status					
Unmarried	40	16.0	53.58 ± 16.88	0.298	0.827
Married	185	74.0	55.77 ± 14.13		
Divorced	9	3.6	56.78 ± 19.82		
Widowed	16	6.4	54.31 ± 12.39		
Fertility status					
Not yet born	47	18.8	54.26 ± 16.31	0.729	0.536
One child	108	43.2	56.82 ± 15.96		
Two children	55	22.0	53.55 ± 12.44		
Three or more children	40	16.0	55.20 ± 11.50		
Family caregiver					
The person himself or herself	186	74.4	55.51 ± 14.88	2.445	0.065
Partner	32	12.8	59.63 ± 12.99		
Parent/Child	21	8.4	48.81 ± 15.18		
Other	11	4.4	53.00 ± 10.48		
Education level					
Elementary school and below	36	14.4	50.69 ± 12.19	2.628	0.035*
Junior high school	85	34.0	53.74 ± 13.51		
High school or junior college	69	27.6	56.20 ± 14.13		
College	38	15.2	60.58 ± 17.02		
Undergraduate and above	22	8.8	57.59 ± 17.52		
Occupational status					
Employed	53	21.2	57.68 ± 16.15	3.327	0.020*
Retired	101	40.4	57.47 ± 13.16		
Unemployed	71	28.4	52.68 ± 15.05		
Freelance	25	10.0	49.56 ± 13.91		
Per capita monthly household income					
≤ 3000	50	20.0	53.48 ± 15.78	0.746	0.525
3001–5000	80	32.0	57.02 ± 13.49		
5001–8000	57	22.8	55.84 ± 15.36		
≥ 8001	63	25.2	54.30 ± 14.60		
Medical payment methods					
Urban and rural residents' medical insurance	74	29.6	52.97 ± 13.93	−1.676	0.095
Employee medical insurance	176	70.4	56.36 ± 14.87		
Number of years on dialysis (years)					
< 1	49	19.6	54.76 ± 12.65	0.068	0.977
1–5	138	55.2	55.70 ± 15.53		
6–10	48	19.2	54.92 ± 14.65		
> 10	15	6.0	55.67 ± 13.66		
Dialysis frequency (times/week)					
2	32	12.8	54.03 ± 12.98	1.235	0.293
3	211	84.4	55.29 ± 14.82		
Other	7	2.8	63.57 ± 16.27		
Kidney transplant experience					
Yes	7	2.8	56.00 ± 6.71	0.243	0.814
No	243	97.2	55.34 ± 14.83		
Type of medicine used (species)					
0	2	0.8	59.50 ± 14.85	0.849	0.495
1	9	3.6	58.22 ± 10.99		
2	23	9.2	54.83 ± 15.87		
3–5	118	47.2	56.80 ± 13.83		
> 5	98	39.2	53.41 ± 15.61		
Type of other diseases in combination (species)					
< 3	141	56.4	56.10 ± 15.14	0.907	0.365
≥ 3	109	43.6	54.40 ± 14.01		

*Note:* Data were judged to be approximately normally distributed using P–P plots.

* *p* < 0.05 indicates statistically significant difference.

### 4.2 Univariate Analysis of the Influencing Factors of BF in MHD Patients

The comparison of BF scores in MHD patients with different genders, occupational statuses and educational levels showed statistically significant differences (*p* < 0.05), as shown in Table 1.

### 4.3 Scores of BF, Social Support and Life Orientation

The total score of BF in MHD patients was (55.36 ± 14.65) points, the total score of social support was (54.41 ± 10.92) points and the total score of life orientation was (19.11 ± 2.62) points. The scores of each dimension and total scale are shown in Table 2.

**TABLE 2:** Scores of BF, social support and life orientation among MHD patients.(*n* = 250).

Item	Number of items	Score range	Score (points, $\bar{x}\pm s$)	Average score of items (points, $\bar{x}\pm s$)
Total BF score	26	17–94	55.36 ± 14.65	2.06 ± 0.57
Spiritual growth	2	0–8	3.44 ± 2.35	1.72 ± 1.17
Appreciation of life and living	5	0–20	11.10 ± 4.14	2.22 ± 0.82
Appreciation of social support	4	1–16	9.20 ± 2.70	2.30 ± 0.67
Personal growth	7	0–28	15.15 ± 5.20	2.16 ± 0.74
Altruistic behaviour	3	0–12	4.96 ± 3.37	1.65 ± 1.12
Health behaviour change	5	2–20	11.49 ± 3.57	2.29 ± 0.71
Total Social Support score	12	12–84	54.41 ± 10.92	4.53 ± 0.91
Family support	4	4–28	22.84 ± 3.54	5.71 ± 0.88
Friend support	4	4–28	14.32 ± 5.67	3.58 ± 1.41
Other support	4	4–28	17.24 ± 4.94	4.31 ± 1.23
Total Life Orientation score	6	0–24	19.11 ± 2.62	13.11 ± 2.62

### 4.4 Analysis of the Correlation Between BF of MHD Patients and Social Support, Life Orientation

The total BF score and its dimensional scores were positively correlated with family support, friend support, other support and life orientation (all *p* < 0.05), as shown in Table 3.

**TABLE 3:** Correlation analysis between BF, social support and life orientation among MHD patients (*n* = 250).

Variable	BF	Spiritual growth	Appreciation of life and living	Appreciation of social support	Personal growth	Altruistic behaviour	Health behaviour change
Social support	0.423***	0.158*	0.385***	0.498***	0.379***	0.129*	0.255***
Family support	0.288***	0.076	0.213***	0.508***	0.233***	0.042	0.224***
Friend support	0.330***	0.190**	0.316***	0.295***	0.284***	0.112	0.155*
Other support	0.349***	0.076	0.337***	0.399***	0.345***	0.127*	0.225***
Life orientation	0.236***	0.121	0.193**	0.134*	0.255***	0.113	0.141*

*Note:* Data were judged to be approximately normally distributed using P–P plots.

* *p* < 0.05 indicates statistical significance.

** *p* < 0.01 indicates statistical significance.

*** *p* < 0.001 indicates statistical significance.

### 4.5 Multiple Linear Regression Analysis of Factors Associated With BF Among MHD Patients

Multiple linear regression analysis was performed to explore influencing factors of BF. Independent variables included gender, education level, occupational status, family support, friend support, other support and life orientation. Categorical variables were numerically coded: gender (male = 1, female = 2); education level (junior high school and below = 1, high school or technical secondary school = 2, college and above = 3); occupational status (employed = 1, retired = 2, unemployed or freelance = 3). Continuous variables including family support, friend support, other support and life orientation were entered into the model with original scores. All variables were incorporated into the regression model. The results showed that gender (female vs. male: *β* = 0.616, *p* < 0.001), education level (*β* = 0.567, *p* < 0.001), occupational status (*β* = 0.479, *p* < 0.001), family support (*β* = 0.114, *p* = 0.003), friend support (*β* = 0.067, *p* = 0.015) and life orientation (*β* = 0.244, *p* = 0.001) were significant predictors of BF. Other support (*β* = 0.056, *p* = 0.090) did not reach statistical significance. Specifically, female gender, higher education level, retired status and higher levels of family support, friend support and life orientation were associated with higher BF scores. Regarding categorical variables, post hoc comparisons using the LSD method further revealed that patients with a college degree or above had significantly higher BF scores compared to those with junior high school or below (*p* < 0.001) and retired patients demonstrated higher BF scores compared to employed patients (*p* < 0.001), as shown in Table 4.

**TABLE 4:** Multiple linear regression analysis of factors associated with BF among MHD patients (*n* = 250).

Variable	Partial regression coefficient	Standard error	Standardized regression coefficient	*t*	*p*
Constant	0.107	0.304	—	0.352	0.725
Gender	0.716	0.019	0.616	38.003	0.000***
Male	0.616	0.037	0.530	16.874	0.000***
Female	0.050	0.024	0.043	2.037	0.043*
Education level	0.567	0.020	0.808	28.854	0.000***
Junior high school and below	0.675	0.040	0.591	16.788	0.000***
High school or junior college	0.240	0.057	0.188	4.243	0.000***
College and above	−0.196	0.038	−0.147	−5.159	0.000***
Occupational status	0.479	0.017	0.631	28.997	0.000***
Employed	0.842	0.057	0.603	14.827	0.000***
Retired	0.345	0.048	0.297	7.161	0.000***
Unemployed or freelance	−0.351	0.032	−0.299	−11.049	0.000***
Social Support	0.215	0.037	0.342	5.866	0.000***
Family support	0.114	0.039	0.177	2.958	0.003**
Friend support	0.067	0.027	0.166	2.444	0.015*
Other support	0.056	0.033	0.12	1.7	0.09
Life orientation	0.244	0.073	0.187	3.340	0.001***

*Note: R* ^*2*^ = 0.251, Adjusted *R* ^*2*^ = 0.236, *F* = 16.392, *p* = 0.000; Data were judged to be approximately normally distributed using P–P plots.

* *p* < 0.05 indicates statistical significance.

** *p* < 0.01 indicates statistical significance.

*** *p* < 0.001 indicates statistical significance.

## 5 Discussion

This study simultaneously examined social support and life orientation in relation to BF among MHD patients, offering new insights into the psychosocial factors associated with BF in this population. This section discusses the key findings in relation to the existing literature, including the current status of BF, the influencing factors and implications for clinical practice.

### 5.1 Status of BF, Social Support and Life Orientation in MHD Patients

The results of this study show that the total BF score of MHD patients was (55.36 ± 14.65), which is at a moderate level. The total score of social support was (54.41 ± 10.92), indicating a moderate level of perceived support and the total score of life orientation was (19.11 ± 2.62), suggesting a relatively optimistic orientation. As no standard cutoff point exists for the BF Scale, the classification of BF as ‘moderate’ in this study is based on a relative comparison with previously published studies in similar populations. Compared to the survey results from Qian et al. (Qian et al. 2024) on chronic disease populations, the BF level in our MHD patients was significantly lower. This may be related to regional cultural differences, insufficient stratification of disease severity and differences in tool application (Li et al. 2025). Similar BF levels have also been reported in recent studies of patients with chronic illness (Liu et al. 2026; Yang et al. 2024), supporting the moderate level observed in our sample. Previous studies have shown that the duration of dialysis in MHD patients is significantly positively correlated with disease acceptance (Zhu, Li, et al. 2024), meaning that as dialysis time increases, patients' cognition and acceptance of the disease gradually improve. The data from this study (Table 1) indicate that 80.4% of patients have been on dialysis for ≥ 1 year (1–5 years: 138 cases; 6–10 years: 48 cases; > 10 years: 15 cases). Patients in the early stages of dialysis, due to insufficient systematic knowledge about the treatment, often experience treatment uncertainty and prognostic anxiety, which may indirectly reduce BF capacity by impairing psychological resilience. In contrast, long‐term dialysis patients improve their disease acceptance significantly by accumulating treatment experience, improving self‐management strategies and establishing stable doctor‐patient support networks (Zhu and Zheng 2021), which in turn promotes an increase in BF levels. Based on these findings, future interventions could explore a structured health education program for new patients with dialysis ≤ 6 months, including: (i) constructing a disease knowledge map (stage‐based popular science manual); (ii) psychological resilience training modules; (iii) a synchronous education plan for family members. Such interventions may help accelerate the patient's adaptive transition and shorten the ramp‐up period of BF.

### 5.2 Factors Influencing BF in MHD Patients

#### 5.2.1 Gender Was Associated With BF

In this study, a correlation was found between gender and BF in MHD patients, with females exhibiting significantly higher BF than males, consistent with previous research findings (Fan et al. 2025). This may be because women pay more attention to their health, are more willing to actively acquire health knowledge and skills through various channels and are also more inclined to express their inner thoughts and needs. They also proactively communicate with healthcare providers, thereby enabling them to cope with their illness more effectively. Therefore, healthcare professionals may consider adopting gender‐specific strategies. For male patients, strengthening health education and communication through diverse media and case sharing may help foster a positive mindset. For female patients, health guidance may be deepened through lectures and training sessions.

#### 5.2.2 Educational Level Was Associated With BF

In this study, MHD patients with higher education levels exhibited significantly better BF levels than those with lower education, which aligns with the findings of Kritikos (Kritikos et al. 2022). One possible reason is the disparity in healthcare resource utilization. Patients with lower educational levels are often constrained by inadequate health literacy, which hinders their ability to effectively access disease management information (Li et al. 2024). As a result, communication with healthcare providers tends to remain superficial, focusing merely on the execution of medical advice. Conversely, highly educated patients build a multidimensional knowledge system by actively searching medical literature and participating in online patient communities, thereby forming a ‘cognitive buffer zone’ to alleviate the stress associated with their illness (Bossola et al. 2024). Another reason might be the combined effect of economic and social capital. Individuals with higher education often enjoy better financial income, allowing them to opt for more flexible treatment plans and reduce conflicts between work and treatment (Bossola et al. 2024). In contrast, those with lower education levels are often compelled by financial constraints to choose low‐cost but less satisfactory treatment options, which exacerbates their sense of treatment fatigue (Li et al. 2024). From the perspective of occupational status, employed patients with stable income may have better access to flexible treatment arrangements and workplace support, whereas unemployed or freelance patients may face greater financial strain and treatment‐related work conflicts, which could further reduce their BF levels (s et al. 2024). For healthcare providers, visual educational toolkits (including illustrated manuals and animated videos) may be considered for the low‐education group, with a focus on visualizing disease progression and training in the breakdown of operational steps. For the high‐education group, a ‘Knowledge Expansion Program’ could be implemented through online case study seminars to deepen their understanding of disease management. Future interventions could also explore structured empowerment programs for MHD patients, such as a phased program covering treatment adherence training, complication prevention skills and quality‐of‐life optimization strategies. In addition, future studies could explore the use of AI‐based tools to provide personalized health reminders and reduce cognitive load in MHD patients. These suggestions are consistent with recent evidence suggesting that tailored educational interventions and structured support programs may help improve self‐management and psychological outcomes among patients with chronic illness (Liu et al. 2026).

#### 5.2.3 Occupational Status Was Associated With BF

In this study, occupational status had a significant negative impact on BF, with patients who were employed with a stable income (employed group) and those who were retired with a pension (retired group) showing significantly higher BF levels than those who were unemployed or had unstable employment (the unemployed/freelance group). The underlying reason may lie in the impact of the disease on labour capacity. Due to the irreversible nature of the illness, MHD patients require long‐term, frequent dialysis, which leads to diminished work ability and reduced quality of life, thereby weakening their level of BF. Although advancements in dialysis technology have improved survival rates (with a 5 year survival rate of approximately 40%) (Flythe and Watnick 2024), patients still need to attend regular medical appointments and take medications, resulting in forced lifestyle changes that often trigger negative emotions such as anxiety and depression, further hindering their return to work. Another possible reason is the financial and medical burden. Long‐term dialysis treatment significantly increases healthcare costs, which, when combined with required dietary restrictions and reduced physical activity capacity, creates a cumulative economic burden that often compels patients to abandon their employment (Bossola et al. 2024). Healthcare professionals may consider offering flexible dialysis scheduling options for working patients and collaborating with employers to establish adaptable work policies. In addition, sharing case studies to strengthen ‘disease adaptability’ training may help patients reconstruct their perception of professional value. For unemployed or freelance patients, future interventions could incorporate narrative therapy or structured diary‐keeping to help them identify positive life events and enhance BF. Future studies may also explore rehabilitation‐to‐employment transition pathways for younger or middle‐aged patients who are willing to return to work, including phased physical assessments and job matching services. For employed patients, brief psychological support and mindfulness‐based stress reduction training may help alleviate workplace anxiety.

#### 5.2.4 Social Support Was Associated With BF

In this study, family support and friend support were positively correlated with BF in MHD patients, consistent with Zhao's (Zhao et al. 2025) research findings. Patients who perceive high levels of social support tend to receive greater support from family and friends, which helps them maintain a positive treatment attitude, avoid negative emotions and enhance their BF levels. The stronger the individual's social support network, the better they can cope with external challenges. Good support helps patients release stress, accept their illness and enhance their sense of control over life (Yao et al. 2022). For patients with low self‐esteem or diminished self‐worth, the support and care of family members are especially critical in fostering a sense of value and belonging. This suggests that healthcare providers may consider assessing patients' social support levels and encouraging family members of those with low support to show more care. Emphasizing family support, regularly assessing family functions and encouraging family members to offer understanding and support may be beneficial. At the same time, providing dialysis education and psychological counselling, promoting communication among family members and encouraging participation in community activities may be helpful (Legrand et al. 2020). Future interventions could also explore the establishment of psychological support platforms, peer support groups and structured communication programs to help patients cope with stress, practice self‐care and strengthen their family and friend networks.

#### 5.2.5 Life Orientation Was Associated With BF

In this study, life orientation was positively correlated with BF in MHD patients, consistent with the findings of Kuchi (Kuchi et al. 2023). Optimistic patients are skilled in communication and sharing, allowing them to gain more information and support, which helps with psychological adjustment during dialysis, overcoming adversity and making positive changes. Therefore, their BF levels are higher (Ma et al. 2023). Optimistic orientation is a protective factor for BF, enabling patients to self‐regulate, actively cooperate with treatment, adjust negative emotions, develop a sense of treatment control and improve quality of life (Paszkowska‐Rogacz 2024). This suggests that healthcare workers may consider paying attention to the optimism of MHD patients. Future studies could explore positive psychological interventions, such as optimism training modules, to enhance patients' experiences with difficulties and guide them to respond to illness positively. In addition, distributing family health guides and organizing self‐care seminars for vascular access may help enhance patients' understanding of health‐related knowledge and further boost their confidence in treatment. Encouraging patients to express their inner feelings actively may help alleviate their psychological burdens, strengthen their confidence in treatment and encourage them to participate more proactively in the disease management process.

### 5.3 Interrelationships Among BF, Social Support and Life Orientation

The correlation analysis revealed that BF was positively correlated with social support (*r* = 0.423, *p* < 0.001) and life orientation (*r* = 0.236, *p* < 0.001), indicating that higher levels of social support and life orientation were associated with higher BF levels among MHD patients. These findings suggest that social support and life orientation may function as interconnected psychosocial resources in promoting positive psychological adaptation among MHD patients. The relationship between social support and BF can be understood through the stress‐buffering model. Patients who perceive adequate support from family, friends and others are more likely to feel understood, valued and cared for, which in turn helps them maintain a positive treatment attitude and enhances their capacity to identify benefits in their illness experience (Fan et al. 2025). Previous studies have confirmed that social support is a key positive predictor of BF in various chronic disease populations (Yang et al. 2024). Similarly, life orientation plays a crucial role in facilitating BF. Optimistic patients tend to adopt more active coping strategies, communicate more effectively with healthcare providers and maintain a more positive outlook on their future, which collectively enables them to derive greater meaning and growth from their illness experience (Von Rezori et al. 2024). Studies have shown that optimism is a protective factor for psychological well‐being, enabling patients to self‐regulate negative emotions and develop a sense of control over their treatment (Zorba et al. 2024). Notably, social support and life orientation may also interact with each other. Patients with higher levels of optimism may be more proactive in seeking and maintaining social support networks, which in turn further enhances their BF levels. This possible synergistic effect warrants further investigation in future studies, particularly regarding whether interventions targeting both social support and life orientation may yield greater benefits than addressing each factor alone. Future studies are warranted to explore the potential mediating or moderating mechanisms among these three variables.

## 6 Conclusion

In summary, BF levels among MHD patients have substantial room for improvement. Gender, educational level, occupational status, family support, friend support and life orientation were identified as independent predictors of BF, while other support did not show a significant association. Specifically, female gender, higher educational level, retired status and higher levels of family support, friend support and life orientation were associated with higher BF scores. Given that BF enables patients to identify positive meanings in their illness experiences, healthcare professionals should prioritise assessing BF among MHD patients, guiding them to foster psychological growth, enhancing their health management skills and ultimately improving their BF outcomes.

## 7 Limitations

The present study has some objective limitations. On one hand, all samples were recruited from large urban hospitals, which introduces a regional bias and limits the generalizability of the findings. Several potential sources of bias should also be acknowledged. First, convenience sampling may have introduced selection bias and sampling bias, as patients who agreed to participate may differ systematically from those who declined and patients treated at large urban hospitals may have better access to healthcare resources and higher health literacy than those in primary or rural settings. Second, response bias cannot be excluded, as self‐reported questionnaires may be influenced by social desirability, recall bias, or misunderstanding of items. These biases may have influenced the observed levels of BF, social support and life orientation, as well as their associations. Future research could focus on MHD patients living in non‐urban areas and adopt multicenter, population‐based, or random sampling designs to reduce these biases. On the other hand, this study is a cross‐sectional study and does not assess the dynamic changes in patients' BF levels. Subsequent studies could employ longitudinal research methods further to explore the trends in BF changes in MHD patients and gain a deeper understanding of its developmental process. Additionally, this study did not include depression as a covariate. Future studies should incorporate depression and other psychological variables to control for potential confounding effects.

## 8 Implications for Nursing Practice

Based on the significant findings of this study, several implications for nursing practice can be drawn. First, given that BF was at a moderate level and was positively associated with social support and life orientation, nurses should routinely assess BF and related psychosocial factors in MHD patients and incorporate these assessments into routine dialysis care. Assessment can be conducted by trained dialysis nurses using brief validated tools such as the BF Scale and the Perceived Social Support Scale, at least once every 3 months or when clinically indicated. Second, because gender, education level and occupational status were significant predictors of BF, nursing interventions should be tailored to patients' demographic and socioeconomic characteristics. These interventions can be delivered by dialysis nurses with training in health education and communication, through brief individual sessions of 20–30 min, once or twice a month and can be integrated into routine dialysis visits. Third, as family support and friend support were significantly associated with BF, nurses should prioritise strengthening family‐centred and peer‐related support networks. This can be achieved through family education sessions, peer support groups and structured communication programmes, delivered by nurses or trained peer leaders, with a frequency of once a month and a duration of 60–90 min per session. Fourth, since life orientation was a significant predictor of BF, nurse‐led positive psychological interventions aimed at enhancing optimism and resilience should be integrated into routine care. These interventions can be delivered by nurses with basic training in positive psychology or resilience‐building, through brief sessions of 30–45 min, once every 2 weeks, with expected outcomes including improved optimism, resilience and BF levels. These implications are directly derived from the significant findings of this study and highlight the strengths of a psychosocially oriented nursing approach in promoting BF among MHD patients.

## Author Contributions

**Xianghao Chen:** conceptualization, investigation, formal analysis, writing – original draft, writing – review and editing. **Xiaodan Lin:** investigation, formal analysis. **Haidan Xie:** investigation, formal analysis. **Jialian Huang:** writing – review and editing. **Huifeng Song:** writing – review and editing. **Xiaozhou Zhou:** conceptualization, methodology, supervision, writing – review and editing, project administration.

## Funding

The authors have nothing to report.

## Ethics Statement

This study was approved by the Research Ethics Committee of the First Affiliated Hospital of Guangdong Pharmaceutical University (Approval No. 2024‐IIT‐47).

## Consent

Written informed consent was obtained from all participants prior to data collection.

## Conflicts of Interest

The authors declare no conflicts of interest.

## Supporting information


**Figure S1:** Participant flow diagram.

**Data S1:** Supporting Information.

## Data Availability

The data that support the findings of this study are available on request from the corresponding author. The data are not publicly available due to privacy or ethical restrictions.
