# Supplementary material for: Benefit Finding in Maintenance Haemodialysis Patients: Associations With Social Support and Life Orientation
Source: Nurs Open. 2026 Oct 28;13(10):e70946. doi: 10.1002/nop2.70946 (PMC13716320; doi:10.1002/nop2.70946)

Supplementary Figure 1. Participant flow diagram

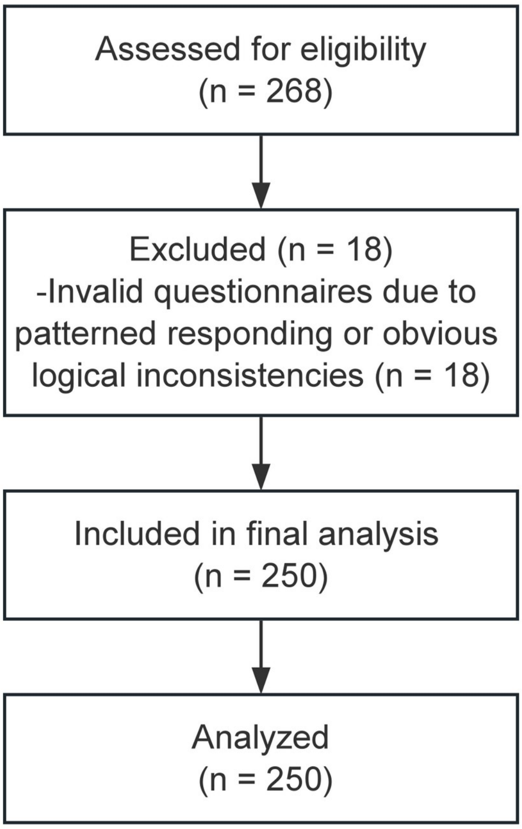

Supplement: Supplementary file 1 [file NOP2-13-e70946-s001.docx]
